# DNA Sensing Platforms: Novel Insights into Molecular Grafting Using Low Perturbative AFM Imaging

**DOI:** 10.3390/s23094557

**Published:** 2023-05-08

**Authors:** Silvia Maria Cristina Rotondi, Paolo Canepa, Elena Angeli, Maurizio Canepa, Ornella Cavalleri

**Affiliations:** 1 Dipartimento di Fisica and Optmatlab, Università di Genova, Via Dodecaneso 33, 16146 Genova, Italy; silviamariacristina.rotondi@edu.unige.it (S.M.C.R.); canepa@fisica.unige.it (M.C.); 2 Dipartimento di Fisica, Università di Genova, Via Dodecaneso 33, 16146 Genova, Italy; elena.angeli@unige.it; 3 INFN, Sezione di Genova, Via Dodecaneso 33, 16146 Genova, Italy

**Keywords:** nanoshaving, nanografting, AFM, quantitative imaging (QI), DNA, hybridization, biosensor

## Abstract

By using AFM as a nanografting tool, we grafted micrometer-sized DNA platforms into inert alkanethiol SAMs. Tuning the grafting conditions (surface density of grafting lines and scan rate) allowed us to tailor the molecular density of the DNA platforms. Following the nanografting process, AFM was operated in the low perturbative Quantitative Imaging (QI) mode. The analysis of QI AFM images showed the coexistence of molecular domains of different heights, and thus different densities, within the grafted areas, which were not previously reported using contact AFM imaging. Thinner domains corresponded to low-density DNA regions characterized by loosely packed, randomly oriented DNA strands, while thicker domains corresponded to regions with more densely grafted DNA. Grafting with densely spaced and slow scans increased the size of the high-density domains, resulting in an overall increase in patch height. The structure of the grafted DNA was compared to self-assembled DNA, which was assessed through nanoshaving experiments. Exposing the DNA patches to the target sequence produced an increase in the patch height, indicating that hybridization was accomplished. The relative height increase of the DNA patches upon hybridization was higher in the case of lower density patches due to hybridization leading to a larger molecular reorganization. Low density DNA patches were therefore the most suitable for targeting oligonucleotide sequences.

## 1. Introduction

The search for increasingly sensitive and reliable methods for the early diagnosis of pathological conditions has stimulated intensive research aimed at developing functional biosensing platforms [1,2]. Large attention has been focused on hybrid interfaces obtained by surface immobilization of probe molecules capable of selectively detecting target biomarkers [3]. Different analytical methods have been exploited to monitor biomarker recognition, including optical detection via surface plasmon resonance (SPR) [4,5,6] or spectroscopic ellipsometry (SE) [7,8,9], electrochemical [10,11,12] or spectrochemical methods [13,14,15,16], mass sensitive methods such as quartz crystal microbalance (QCM) [17,18] or nanomechanical resonators [19], and AFM-based nanolithography methods [20,21,22,23,24].

In recent years, we have developed a multi-technique analytical approach to evaluate the sensing properties of hybrid interfaces by coupling AFM, SE, EIS, and QCM [7,25,26,27,28]. We focused on the study of self-assembled monolayers (SAMs) of thiol-terminated molecules on gold, from simple alkanethiols to DNA strands and proteins. The combined use of multiple analytical methods [25,28,29,30,31,32] which probe different interfacial properties, is particularly effective for analyzing ultrathin organic films, as it increases the degree of confidence in the obtained results.

AFM is a well-suited method for the analysis of ultrathin, flat samples such as SAMs on ultraflat gold. Following the pioneering work of Liu [21,33], it became clear that AFM can be used not only as a high-resolution imaging tool, but also as a nanolithography tool capable of patterning ultrathin organic films at the nano/micrometer scale [20]. In nanoshaving mode, the tip of the AFM works as a shaver capable of removing molecules from selected areas of the sample. We previously applied this method to SAMs of alkanethiols [26], amino phosphonates [34,35], thiolated DNA [36], and protein films [37] to evaluate the thickness of organic films by studying the difference in height between the film and the bare substrate where the molecules were removed. In the multi-technique approach we have developed, the AFM evaluation of the film thickness is an important point for the analysis of the correlated optical data. In a previous study of SAMs of alkanethiols bearing ethylene glycol groups as end-groups, the combined AFM and SE analysis allowed us to evaluate the vertical density profile of the SAM related to the degree of hydration of the film [26]. Combined AFM nanoshaving and SE analysis allowed for evaluation of the effect of buffer ionic strength on the density of DNA SAMs [28]. Furthermore, by comparing AFM, SE, and QCM measurements, we could monitor the hybridization process of surface-immobilized DNA and provide evidence of DNA hypochromism in DNA SAMs [7].

Nanografting represents a step forward towards the use of AFM as a nanolithography tool. In this case, the AFM tip is scanned over a thiolated SAM in the presence of another thiol molecule. The high force applied by the tip allows for the exchange of thiolated molecules between the surface and the solution over the scanned regions, thus leading to the formation of patterned SAMs. Nanografting was initially applied to alkanethiol SAMs [33,38], and has been successfully exploited to produce DNA nano/micropatterns in alkanethiol SAMs [20,21,39,40,41,42,43,44]. Selective hybridization of the nanografted regions with complementary DNA has been used to anchor functional peptides/proteins to the nanografted platforms by exploiting DNA-directed immobilization [22,23,24,45].

Several reports have focused on the influence of the grafting parameters on the structure of the nanografted patches, showing that the patch height, and hence its molecular density, can be tuned by modulating the molecular concentration of the grafting solution, the tip load, and the density of scan lines [21,24,42,46,47,48]. Following Mirmomtaz et al. [41], the latter parameter can be described by the S/A ratio, where S is the scanned area and A is the actual area of the final patch. This ratio can be expressed as S/A = R·N/L, where R is the radius of the contact region and N is the number of scan lines (in the slow scan direction) divided by the length of the patch L (in the same direction). In this scheme, S/A = 1 indicates that the nanografted lines do not overlap with each other, while S/A = n indicates that each spot in the nanopatch has been nanografted n times over.

In the present work, we investigated the combined effect of the tip scan rate and S/A ratio on both the grafting process and the hybridization of grafted DNA patches. After grafting, we operated the AFM in the quantitative imaging (QI) mode [49]: a non-perturbative mode based on force spectroscopy. In QI, a force–distance curve is acquired in each pixel. By avoiding dragging forces, QI greatly reduces the risk of deformation of the organic film during the measurement, thus allowing reliable evaluation of its thickness. By analyzing QI images, we were able to observe the coexistence of molecular domains of different heights, and thus different densities, within the grafted areas. Decreasing the tip scan rate and increasing the S/A ratio led to an increase in high-density domains within the grafted area, resulting in an overall increase in patch height. The structure of grafted DNA was compared to that of the self-assembled DNA assessed through nanoshaving experiments. 

The experience gained from producing and characterizing grafted interfaces will be utilized in future experiments, in combination with spatially resolved SE, to develop a multiplexing platform for the optical detection of different nucleotide sequences.

## 2. Materials and Methods

### 2.1. Materials

#### 2.1.1. Chemicals

HPLC purified 22-mer ssDNA sequences (thiolated probe sequence: HS-(CH2)6-5′-CTTCACGATTGCCACTTTCCAC-3′ (pDNA), designed to be fully complementary to the target sequence, and target sequence: 5′-GTGGAAAGTGGCAATCGTGAAG-3′ (tDNA)) were purchased from Biomers (Ulm, Germany) and used as received.

Tris[hydroxymethyl]amino-methane (Tris base), ethylenediaminetetraacetic acid (EDTA), and 6-Mercapto-1-hexanol (HS–(CH2)6–OH, MCH) were purchased from Sigma Aldrich (St. Louis, MO, USA). Sodium chloride (NaCl) was purchased from Merck (Darmstadt, Germany).

All DNA and MCH solutions were prepared using ultrapure water from Millipore (resistivity 18.2 MΩ·cm). The experiments were performed in TE buffer (10 mM Tris, 1 mM EDTA, 1 M NaCl, pH adjusted to 7.2 using HCl (Fluka, Buchs, Switzerland)). EDTA was used to chelate metal cations, such as Mg^2+^, which are involved in the activation of DNase [50]. The presence of EDTA blocks DNase activity, preventing DNA degradation.

#### 2.1.2. AFM Gold Substrate Preparation

Ultraflat gold substrates (RMS roughness = 0.3 nm in 2 μm × 2 μm scans), also known as Ulman-type gold, were prepared following the protocol described by Gupta et al. [51]. Ulman substrates do not require cleaning procedures because the capping silicon slide is removed from the gold surface immediately prior to exposure to the self-assembling solution.

### 2.2. Methods

#### 2.2.1. Nanoshaving and Nanografting

For nanoshaving measurements, samples were prepared according to the following three-step deposition protocol: 3 h immersion in a 1 μM solution of pDNA in TE buffer, rinsing in TE buffer, 1 h immersion in a 5 μM solution of MCH in TE buffer, rinsing in TE buffer, 1 h immersion in 1 μM solution of tDNA in TE buffer for hybridization, and rinsing in TE buffer. Nanoshaving experiments were performed after each deposition step in at least six areas per sample to ensure sample uniformity.

For nanografting experiments, gold substrates were first soaked overnight in a 300 μM MCH solution to ensure the deposition of a MCH SAM. ssDNA grafting was performed in a 5 μM pDNA solution, and hybridization was performed using a 50 nM tDNA solution. Nanografting was performed in triplicate for each S/A and scan rate condition.

#### 2.2.2. Atomic Force Microscopy

**Figure 1:**
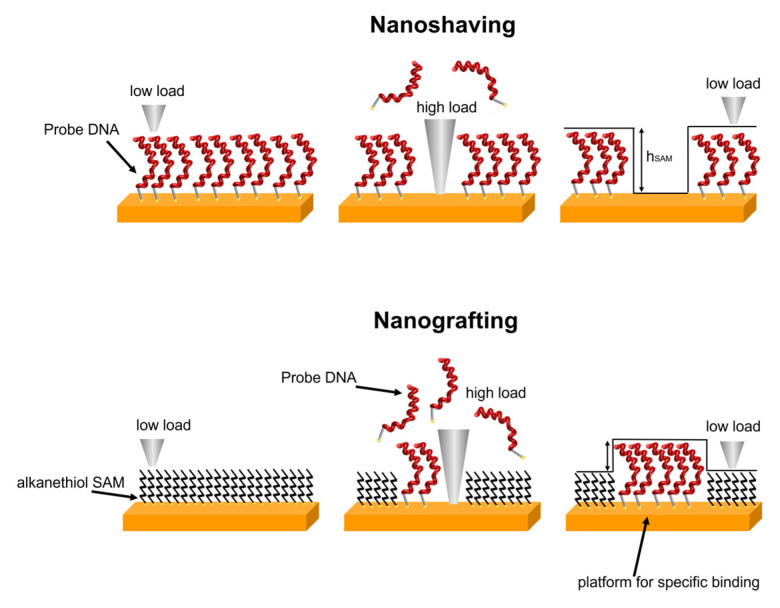
Schematic of nanoshaving and nanografting.

AFM measurements were performed using a JPK Nanoscope IV microscope, Bruker (Billerica, MA, USA) in TE Buffer. Nanoshaving and nanografting experiments were performed in contact mode using aluminium coated silicon cantilevers (OTESPA-R3, Bruker) with a nominal elastic constant of 26 N/m and a nominal tip curvature radius of 10 nm. A force of a few hundred of nN was applied to remove the chemisorbed molecules (for shaving) and to replace MCH with pDNA molecules (for grafting) over selected regions that were a few micrometers in size. A schematic of nanoshaving and nanografting is reported in Figure 1.

Imaging on larger areas was then performed in quantitative imaging (QI) mode using gold-coated silicon cantilevers (DNP-10, Bruker) with a nominal elastic constant of 0.24 N/m and a nominal tip curvature radius of 20 nm. QI mode is an imaging mode based on force spectroscopy. A force–distance curve was acquired for each pixel, avoiding dragging forces and preventing tip and sample damage. By measuring the z position of the tip at a specific force load, it was possible to obtain both topography data and information on the mechanical properties of the sample. 

The data were analyzed using Gwyddion (v2.61) and JPKSPM data processing software (v7.0.162).

## 3. Results and Discussion

**Figure 2:**
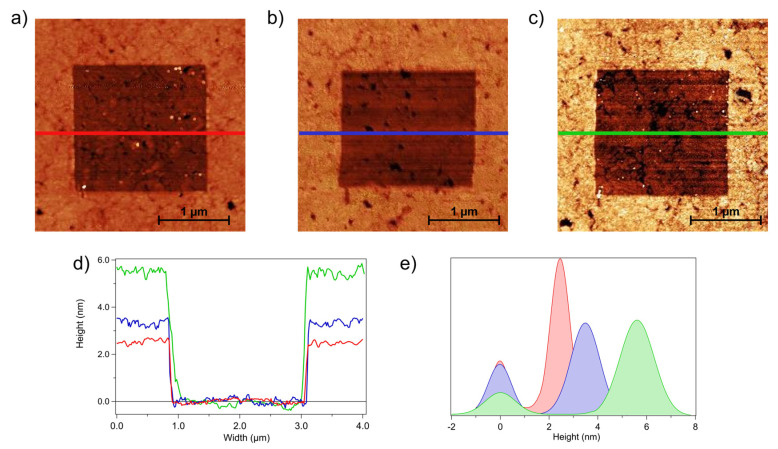
QI AFM images of shaved areas of: (**a**) pDNA, (**b**) pDNA + MCH, and (**c**) dsDNA + MCH (data scale = 13 nm). (**d**) Height profiles obtained along the colored lines, and (**e**) height histograms of the three steps: pDNA in red, pDNA + MCH in blue, and dsDNA + MCH in green. The zero level was set at the gold surface.

As a reference for the comparative analysis of grafted DNA patches and self-assembled DNA SAMs, we performed nanoshaving experiments on pDNA SAMs. Figure 2 shows QI AFM images acquired after nanoshaving on a pDNA SAM before (Figure 2a) and after exposure to MCH (Figure 2b) and to tDNA (Figure 2c). After removal of chemisorbed molecules on the selected area using hard contact scans, the gold substrate was exposed (darker regions in Figure 2a–c), thus allowing the thickness of the DNA film (the surrounding brighter region) to be measured. Representative z profiles along horizontal sections of the images are shown in Figure 2d, where the profiles were aligned by setting the zero at the gold surface. Figure 2e shows the height histograms of the images in Figure 2a–c. For each histogram, the peak centred at 0 nm refers to the gold substrate, and the second peak is related to the SAM. For each histogram, the distance between the two peaks provides the thickness of the film.

The SAM thickness values measured after the three deposition steps are reported in Table 1. The errors were calculated as the semi-difference between the highest and the lowest measured values.

These results indicate that significant molecular reorganization occurred after each deposition step. The film thickness measured after the pDNA deposition step was significantly smaller than the thickness expected for a SAM of extended pDNA strands upright on the surface (a total molecular length of 8.5 nm would be expected, considering a length of 0.34 nm per base [52] and a length of 1 nm for the thiolated linker, determined using nanoshaving experiments [14], see Section S1). At a high ionic strength, as was the case in the present study, the persistence length of ssDNA is approximately 10 Å [53]. A partially folded conformation was therefore expected for pDNA, which explains the low film thickness. Moreover, due to the significant interaction between nitrogenous bases and the gold surface [54,55] some pDNA strand may have been partially lying on the surface. Introducing MCH improves the film organization [56] by partially reorienting the pDNA strands, as inferred from the increased film thickness. Finally, hybridization with tDNA helps to stretch the chains and organize them on the surface, leading to a further increase in the film thickness. We note that the dsDNA film thickness was shorter than the extended molecular length, implying that the chains were not completely upright and straightened.

An estimation of the surface molecular density can be derived from the SAM height using a simple molecular model [21]. We modeled the pDNA and dsDNA as cylinders with a height of 8.5 nm (see above), a diameter of 1.7 nm for ssDNA [57], and a diameter of 2.9 nm for dsDNA [58] (in both cases, the diameter was larger than the geometrical one to account for the contribution of counterions and hydration water). We estimate a pDNA density of approximately 1.4 × 10^13^ molecules/cm^2^ (see Section S2 for details), which is in agreement with the literature [59,60,61]. Using the same model, a hybridization efficiency of approximately 45% was estimated based on the height of the dsDNA film, in agreement with the result we obtained from QCM-D analysis on another 22-mer oligonucleotide sequence [7].

**Figure 3:**
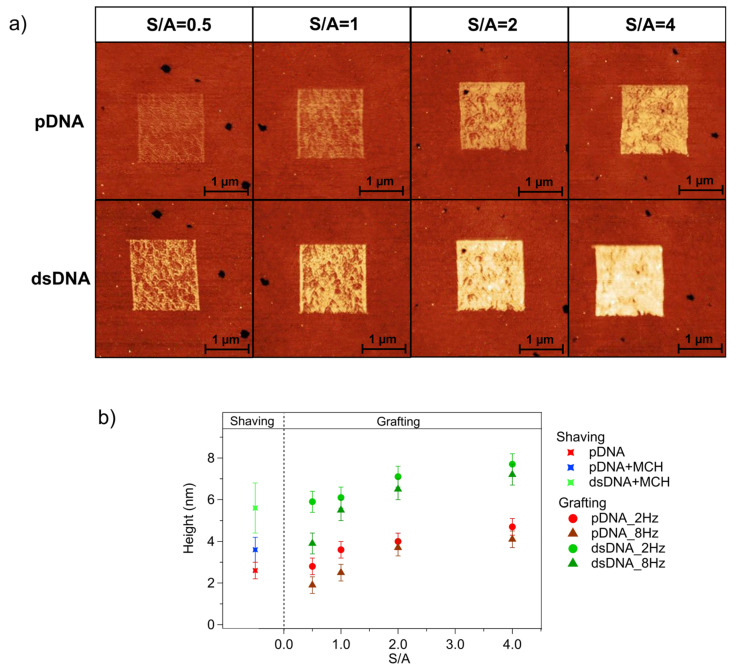
(**a**) QI AFM images of: (upper row) pDNA patches grafted with increasing S/A (from left to right S/A = 0.5, S/A = 1, S/A = 2 and S/A = 4) at scan rate = 8 Hz; (lower row) same patches after hybridization with 50 nM tDNA.Data scale = 13 nm. (**b**) Height (referred to the gold surface) vs. S/A of pDNA patches grafted at a 2 Hz scan rate (dots) and 8 Hz scan rate (triangles), before (red) and after (green) hybridization with tDNA. SAM height values derived from shaving are reported with a cross for comparison. Error bars were calculated as the semi-difference between the maximum and the minimum height values of patches grafted using same scan parameters.

Denser molecular packing can be achieved through grafting experiments. Figure 3 shows the results of a typical nanografting experiment. The brighter squares correspond to the regions where pDNA was grafted into the MCH SAM (the darker surrounding region). pDNA patches were grafted using different tip scan rates and different S/A ratios [41]. We grafted 1.5 μm-wide patches with scan rates of 2 Hz and 8 Hz (corresponding to 6.2 μm/s and 27.5 μm/s tip velocities, respectively) and the following values for the S/A parameters: 0.5, 1, 2, and 4. The AFM images in the upper row of Figure 3a show the pDNA patches grafted with the different S/A values at a scan rate of 8 Hz (see Figure S2 for the corresponding results obtained at 2 Hz).

Visual examination of AFM images indicated that the S/A ratio had a significant effect on the height of the pDNA patches. Larger height differences between the pDNA patch and the surrounding MCH SAM were observed for higher S/A ratios. For a more quantitative evaluation, we derived the height of the pDNA patches relative to the MCH film by analyzing the height histograms. The graph in Figure 3b reports the heights of the pDNA patches grafted at 2 Hz (red circles) and 8 Hz (red triangles) as a function of S/A. The height values reported in Figure 3b represent the total patch height relative to the gold surface, calculated as the sum of the measured patch height and the 1 nm thickness of the MCH SAM (derived from nanoshaving experiments, see Section S1). The plot indicates that the pDNA patch height increased with the S/A ratio. Since the S/A value is related to the spatial density of the scan lines, it is reasonable to assume that a larger amount of MCH molecules is displaced at higher S/A ratios and thereafter replaced by pDNA, thus leading to denser pDNA patches. The scan rate also influences the patch height, although to a lesser extent. For each S/A value, the patches grafted at 2 Hz were taller than the ones grafted at 8 Hz, indicating that lower scan rates resulted in more efficient molecular grafting.

The combined analysis of nanoshaving and nanografting experiments allowed us to compare the height of grafted pDNA patches with the thickness of self-assembled pDNA films. At low S/A ratios, grafted and self-assembled pDNA samples had a similar thickness, while patches grafted at high S/A ratios were thicker than DNA SAMs. As first reported for alkanethiols [62], nanografting creates a spatially confined, transient environment, which accelerates the kinetics of the self-assembly and guides the thiolated molecules towards high-density packing. The higher the S/A ratio, the more efficient the DNA grafting, and the higher the molecular density. The molecular densities of the grafted pDNA derived from the patch height [21] are reported in Table S1 as a function of the grafting conditions. At high S/A ratios, the nanografting produced DNA patches that were thicker and denser than DNA SAMs, as reported in the literature for closely related systems [41].

**Figure 4:**
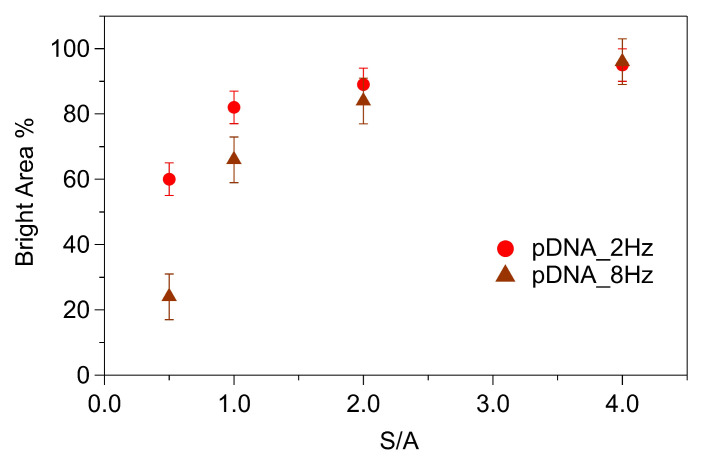
Bright area percentage of DNA patches as a function of S/A. Error bars were calculated as the semi-difference between the maximum and minimum values for patches grafted using the same scanning conditions.

The height values discussed so far were the average values derived from the height histogram analysis. This approach, in line with previous nanografting experiments on related systems [20,21,42], focuses on the average height of grafted patches. Indeed, the analysis of the AFM images acquired in the low-perturbative QI mode revealed the coexistence of different molecular domains within the DNA patches, providing novel insights into DNA grafting. Darker, i.e., thinner, domains, 0.5 nm ÷ 0.7 nm in height (i.e., 1.5 nm ÷ 1.7 nm, referred to the gold surface), coexisted with brighter, i.e., thicker domains. The height of the brighter domains increased from ~2.5 nm for S/A = 0.5 to ~5 nm for S/A = 4 (i.e., from 3.5 nm to 6 nm, referred to the gold surface). The dark domains corresponded to regions characterized by low density DNA grafting into the MCH SAM, where loosely packed DNA strands were randomly lying on the MCH film. Conversely, the bright domains corresponded to regions where the DNA had been more densely grafted, with the grafting efficiency increasing with an increasing S/A ratio. The percentage of brighter areas, i.e., the relative coverage of thicker domains, increased with the S/A ratio, as shown in Figure 4. The bright area percentage was comparatively higher at lower scan rates, confirming that the grafting efficiency was higher at lower scan rates. The difference between the values obtained at the two scan rates vanished when the S/A ratio increased. This behaviour suggests that, although grafting at high scan rates was less efficient than slow grafting, increasing the S/A ratio promotes the grafting process and compensates for the low yield of fast grafting.

A comparison of grafted DNA patches (Figure 3a) with DNA SAMs (Figure 2a) emphasizes the higher uniformity of self-assembled DNA compared to grafted DNA layers. This finding is likely related to the different mechanism of DNA deposition in the two cases. While self-assembled DNA layers represent an equilibrium state reached after a relatively long incubation, grafted DNA layers are the result of an out-of-equilibrium, kinetically controlled process that is driven by the application of an external force from the scanning tip [62,63]. As reported in the literature for alkanethiols, in the case of nanografting, the kinetics of self-assembly are much faster than those associated with the ‘classical’ equilibrium self-assembly on uniform surfaces [38]. The origin of the low uniformity of grafted patches resides in the complexity of the process, which is the result of the subtle interplay between kinetically controlled factors and surface heterogeneity. Structural defects in the underlying gold surface could lead to heterogeneity in the MCH SAM structure. Loosely bound MCH molecules could be more easily removed by the scanning tip, thus favoring DNA grafting. The kinetically controlled rate of molecular replacement could explain the observed dependence of the grafting efficiency on the scan rate and the S/A ratio.

After we characterized the grafted DNA patches, we focused on hybridization. The AFM images in the lower row of Figure 3a were acquired over the corresponding patches of the upper row after exposure to a 50 nM solution of complementary tDNA. The comparison of the AFM images in the upper and lower rows clearly shows an increase in the patch height, indicating successful hybridization with tDNA. The average patch heights (referred to the gold surface) after hybridization are reported in Figure 3b (green symbols) as a function of S/A for both scan rates. Similar to what was observed for pDNA patches, the height of dsDNA patches increased when the S/A value used for pDNA grafting increased, with higher values observed for patches grafted at slower scan rates. Upon hybridization, pDNA patches grafted at S/A = 4 reached a total height between 7 nm and 7.5 nm depending on the grafting scan rate. These height values suggest that, upon hybridization, densely grafted pDNA reaches a stretched configuration, with dsDNA helices oriented almost perpendicular to the surface. Hybridization also led to a significant height increase for less densely grafted ssDNA patches obtained for lower S/A values. However, in these cases, less dense dsDNA helices likely adopted a more tilted orientation. Comparable height increases were observed in ancillary experiments carried out using higher (up to 1 µm) tDNA concentrations, suggesting a saturation in the hybridization efficiency at a 50 nM target concentration. On the other hand, preliminary experiments at lower tDNA concentrations showed lower height increases upon exposure to tDNA. Future experiments will focus on the signal to concentration correlation to define the system LOD.

Other experiments performed using a non-target sequence did not show an increase in the height of the pDNA-grafted patches, confirming that the height increase measured after exposure to tDNA was due to hybridization and not to nonspecific adsorption.

It is worth noting that, although lower S/A values corresponded to lower DNA densities, the relative increase in patch height following hybridization was higher at low S/A ratios. Table 2 reports the hybridization-induced relative increase in the patch height, referred to as the MCH SAM, as a function of S/A and scan rate:

$$percentage\;increase\;\left(\%\right)=\frac{h_{dsDNA}-h_{pDNA}}{h_{pDNA}}\times 100$$

The larger relative increase in patch height measured at low S/A ratios indicates that hybridization resulted in a larger molecular reorganization for patches grafted at a low density compared to denser and more ordered pDNA patches grafted at a high S/A ratio, in agreement with previous reports on similar systems [24].

**Table 2:** Relative percent increases in the patch height upon exposure to complementary DNA as a function of S/A. Associated errors are 15% of the reported values.

	2 Hz	8 Hz
S/A = 0.5	180	230
S/A = 1	120	200
S/A = 2	100	110
S/A = 4	80	100

**Figure 5:**
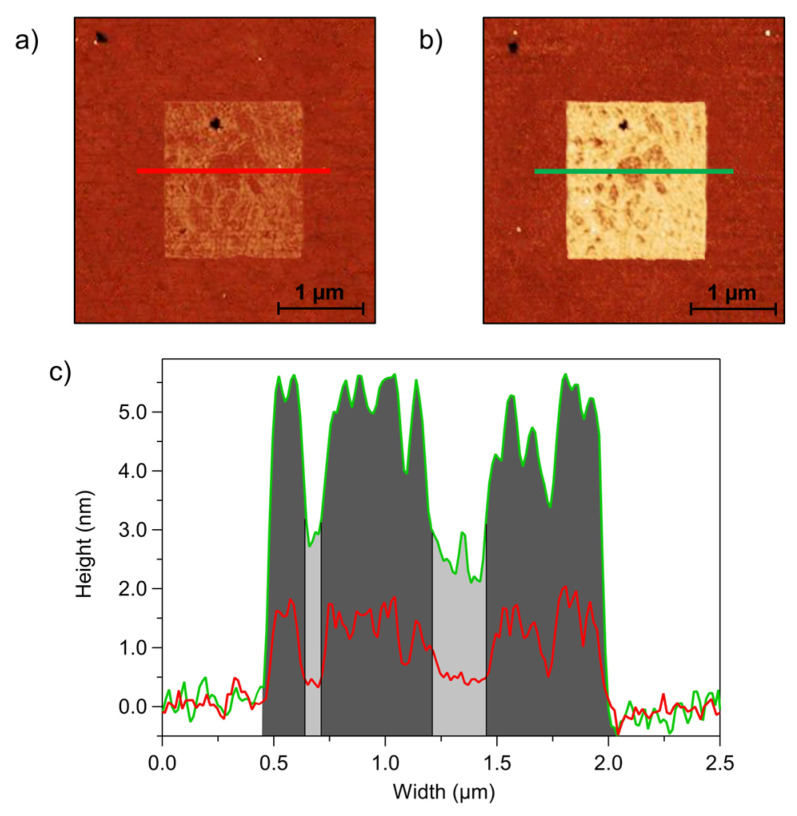
QI AFM images of DNA patches grafted with S/A = 0.5 and scan rate = 2 Hz: (**a**) ssDNA and (**b**) dsDNA. (**c**) Height profiles along the coloured horizontal lines: the red profile refers to ssDNA, and the green profile refers to dsDNA. Regions filled in dark grey indicate higher density DNA areas, and light grey regions refer to lower-density domains.

These considerations regarding the pDNA reorganization upon hybridization derived from the height analysis averaged on the entire patches can be substantiated by the spatially resolved analysis on individual patches as shown on the example of Figure 5. The images in Figure 5 show a pDNA patch grafted at 2 Hz, with S/A = 0.5. A careful comparison of AFM images acquired before (Figure 5a) and after (Figure 5b) exposure to tDNA shows a co-localization of brighter (and darker) regions before and after hybridization. The z profiles of the red (before hybridization) and green (after hybridization) lines (Figure 5c) show that the regions inside the patch that were lower in height before hybridization were also lower after hybridization. However, their relative height increase was larger than the relative increase of higher regions, which was reasonably related to the higher molecular reorganization following hybridization of low-density pDNA strands. This finding is in agreement with previous works [24,41] that report a negligible height increase upon hybridization for densely packed DNA patches, in which pDNA is characterized by an elongated and ordered molecular structure which undergoes minor reorientation upon hybridization. 

Several reports in the literature have addressed the issue of hybridization of immobilized pDNA. Previous SPR [64] and electrochemical [59] investigations examined the dependence of the hybridization efficiency on the pDNA coverage in uniform self-assembled DNA SAMs. More recent studies have focused on grafted DNA patches in which higher DNA packing densities could be achieved compared to self-assembled DNA SAMs [20,21,42]. Hybridization of DNA SAMs confined to arrays of oscillating micrometer-sized pillars was addressed using optical detection methods [65]. Although it is not straightforward to directly compare the hybridization efficiency of the different systems due to the differences in the experimental protocols employed, a general consensus emerges that there is an upper limit to the efficiency of hybridization, which is intrinsically limited by molecular and electrostatic crowding [66].

Following the previous reports and based on our observations (see Table 2), we can conclude that low-density patches grafted at lower S/A ratios were better suited than high density patches for tDNA recognition when the patch height was selected as the indicator of target sequence recognition; in fact, low density patches underwent a larger relative height increase upon hybridization.

## 4. Conclusions

Through a combined nanoshaving and nanografting approach, we investigated the structure of micrometer-sized DNA patches grafted at different S/A ratios and scan rates and compared them with self-assembled DNA films. Tuning the grafting conditions allowed us to tailor the molecular density of the grafted DNA. Employing the low perturbative quantitative imaging mode, we observed the coexistence of molecular domains of different heights, and thus different densities, within the grafted areas. Thinner domains corresponded to low-density DNA regions characterized by loosely packed, randomly lying DNA strands, while thicker domains corresponded to denser DNA patches that were significantly denser than the self-assembled DNA layers. Grafting with densely spaced and slow scans increased the size of the high-density domains, resulting in an overall increase in the patch height.

Exposing the DNA patches to a 50 nM target sequence solution produced an increase in the patch height, indicative of successful hybridization. The relative height increase of the DNA patches upon hybridization was higher in the case of lower density patches since, in this case, hybridization led to a larger molecular reorganization. Low density DNA patches were therefore the most suitable for target sequence detection. Preliminary results showed a measurable increase in the height of low density DNA patches after exposure to target DNA solutions with concentrations as low as 1 nM.

Careful tuning of the grafting conditions is prospectively interesting for successful targeting of short oligonucleotide sequences. The experience gained in the production and characterization of grafted interfaces will be utilized in combination with spatially resolved SE in future experiments. We previously reported the application of SE analysis for uniform DNA-sensing interfaces for the specific and reversible detection of target sequences [7]. As a further development, the miniaturization of the sensing platforms, combined with spatially resolved SE, will help us to develop a multiplexing platform for the optical detection of different oligonucleotides, thus allowing for parallel detection of reduced amounts of target sequences.

## Figures and Tables

**Table 1:** Thickness values obtained from shaving experiments for different SAM deposition steps.

	pDNA	pDNA + MCH	dsDNA + MCH
**Thickness (nm)**	2.6 ± 0.4	3.6 ± 0.6	5.6 ± 1.0

## Data Availability

The data presented in this study are available from the corresponding author upon request.

## Supplementary Materials

The following supporting information can be downloaded at: https://www.mdpi.com/article/10.3390/s23094557/s1, Section S1: MCH Shaving; Section S2: Molecular Density Evaluation of DNA SAMs; Section S3: DNA Nanografting at 2 Hz Scan Rate. Figure S1: (a) AFM height image of shaved MCH SAM (data scale = 4 nm). (b) Height profile obtained averaging horizontal lines that include the shaved area. Zero is set at the gold level. Figure S2: Upper row: QI AFM images of pDNA patches (upper row) obtained at different S/A (from left to right S/A = 0.5, S/A = 1, S/A = 2 and S/A = 4) and scan rate = 2 Hz. Lower row: QI AFM images of the same patches after hybridization with 50 nM tDNA. Data scale = 13 nm. Table S1: Estimated molecular density (molecules/cm^2^) as a function of scan rate and S/A.
