# Supplementary material for: DNA Sensing Platforms: Novel Insights into Molecular Grafting Using Low Perturbative AFM Imaging
Source: Sensors (Basel). 2023 May 8;23(9):4557. doi: 10.3390/s23094557 (PMC10181596; doi:10.3390/s23094557)
Supplement: Supplementary file 1 [file sensors-23-04557-s001.zip › sensors-2362111-supplementary.pdf]

sensors

MDPI

Supporting Information

DNA Sensing Platforms: Novel Insights into Molecular Grafting using Low Perturbative AFM Imaging

Silvia Maria Cristina Rotondi [1], Paolo Canepa [1,*], Elena Angeli [2], Maurizio Canepa [1,3], Ornella Cavalleri [1,*]

1 Dipartimento di Fisica and Optmatlab, Università di Genova, Via Dodecaneso 33, 16146 Genova, Italy; silviamariacristina.rotondi@edu.unige.it (S.M.C.R.); canepa@fisica.unige.it (M.C.)
2 Dipartimento di Fisica, Università di Genova, Via Dodecaneso 33, 16146 Genova, Italy; elena.angeli@unige.it
3 INFN, Sezione di Genova, Via Dodecaneso 33, 16146 Genova, Italy
* Correspondence: paolocanepa@unige.it (P.C.); ornella.cavalleri@unige.it (O.C.)

S1. MCH Shaving

Figure S1 shows the result of a shaving experiment on a MCH SAM. Gold samples were incubated overnight in a 300μM solution of MCH, to ensure de deposition of a MCH SAM. The height of the SAM is obtained from mediating different profiles and is equal to (1.0 ± 0.4) nm.

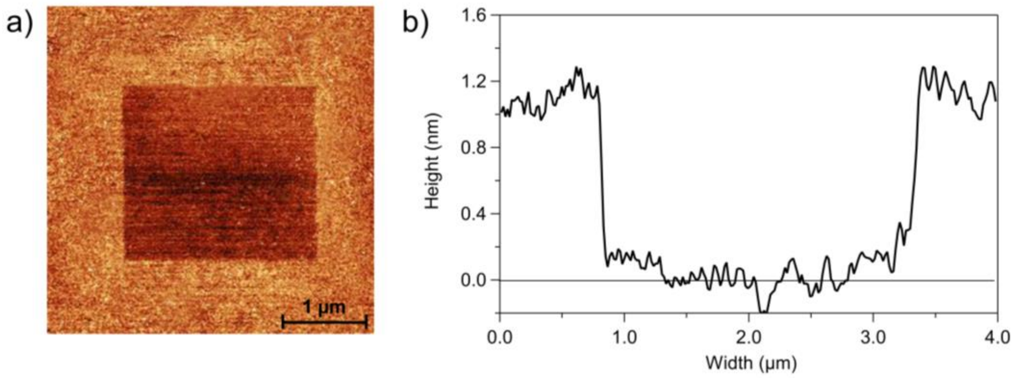

Figure S1. (**a**) AFM height image of shaved MCH SAM (data scale=4nm). (**b**) Height profile obtained averaging horizontal lines that include the shaved area. Zero is set at the gold level.

S2. Molecular Density Evaluation of DNA SAMs

To estimate the molecular density of the SAM and the consequent hybridization we model pDNA and dsDNA as cylinders, with a radius of 0.85 nm [1] and 1.45 nm [2], respectively, to consider electrostatic repulsion and water hydration contribution. Each molecule has a volume given by $V_{mol}=\pi r^2_{mol} h_{mol}$, where h_{mol} is the height of the stretched molecule (7.5nm plus 1nm for the thiol linker).

To evaluate the surface molecular density of pDNA (σ_{pDNA}), we calculate the number of pDNA molecules per unit area A (n_{mol}/A) as the volume per unit area A of the pDNA film V_{pDNA}/A divided by the molecular volume V_{mol}. Therefore:

$$\sigma_{pDNA} = \frac{n_{mol}}{A} = \frac{V_{pDNA}/A}{V_{mol}} = \frac{h_{meas,pDNA}}{\pi r_{mol}^2 h_{mol,ss}}$$

where $V_{pDNA}=A\cdot h_{meas,pDNA}$ and $h_{meas,pDNA}$ is the pDNA SAM thickness measured from shaving experiments.

For dsDNA film we estimate the hybridization efficiency ε, considering that the final dsDNA film will be characterized by a surface density $\varepsilon \cdot \sigma_{pDNA}$ of dsDNA molecules and $(1-\varepsilon) \cdot \sigma_{pDNA}$ of pDNA molecules:

$$V_{dsDNA} = \mathrm{h}_{meas,dsDNA}\mathrm{A} = \varepsilon\sigma_{pDNA}V_{mol,dsDNA}A + (1-\varepsilon)\sigma_{pDNA}V_{mol,pDNA}A$$

Therefore:

$$\varepsilon = \frac{h_{meas,dsDNA} - \sigma_{pDNA}V_{mol,pDNA}}{\sigma_{pDNA}(V_{mol,dsDNA} - V_{mol,pDNA})}$$

S3. Molecular Density Evaluation of DNA Grafted Patches

Using the same geometrical model described in section S2 to evaluate the molecular density of self-assembled DNA films, we estimated the pDNA molecular density in DNA grafted patches. The obtained data are presented in Table S1. Results are in agreement with the literature [3].

Table S1. Estimated molecular density (molecules/cm^2) as a function of scan rate and S/A.

	**2 Hz**	**8 Hz**
**S/A = 0.5**	1.4×10^{13}	9.7×10^{12}
**S/A = 1**	1.7×10^{13}	1.3×10^{13}
**S/A = 2**	2.1×10^{13}	1.9×10^{13}
**S/A = 4**	2.5×10^{13}	2.2×10^{13}

S4. DNA Nanografting at 2 Hz Scan Rate

Figure S2 reports QI AFM images of grafted DNA patches obtained with a scan rate of 2Hz at four different S/A values (0.5, 1, 2 and 4), before (upper row) and after (lower row) hybridization with tDNA. As for patches obtained with a scan rate of 8Hz (Figure 3a) increasing the S/A parameter results in an increase in the patch height, as reported in Figure3b.

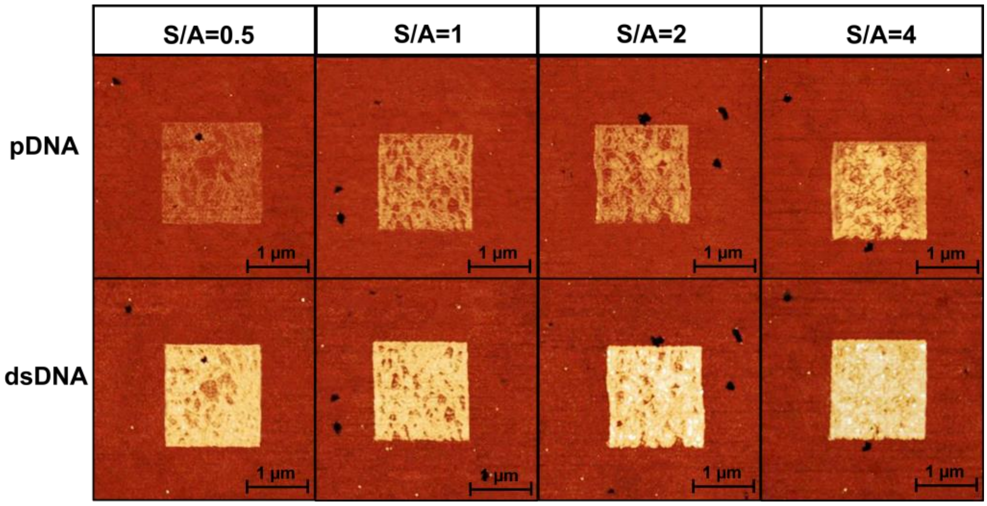

Figure S2. Upper row: QI AFM images of pDNA patches (upper row) obtained at different S/A (from left to right S/A=0.5, S/A=1, S/A=2 and S/A=4) and scan rate=2Hz. Lower row: QI AFM images of the same patches after hybridization with 50nM tDNA. Data scale=13nm.

References

1. Rybenkov, V.V.; Cozzarelli, N.R.; Vologodskii, A.V. Probability of DNA Knotting and the Effective Diameter of the DNA Double Helix. *Proc. Natl. Acad. Sci. U.S.A.* **1993**, *90*, 5307–5311, doi:10.1073/pnas.90.11.5307.
2. Stigter, D. Interactions of Highly Charged Colloidal Cylinders with Applications to Double-Stranded DNA. *Biopolymers* **1977**, *16*, 1435–1448, doi:10.1002/bip.1977.360160705.
3. Bosco, A.; Bano, F.; Parisse, P.; Casalis, L.; DeSimone, A.; Micheletti, C. Hybridization in Nanostructured DNA Monolayers Probed by AFM: Theory versus Experiment. *Nanoscale* **2012**, *4*, 1734, doi:10.1039/c2nr11662f.
